# Identification, Expression, and Roles of the Cystine/Glutamate Antiporter in Ocular Tissues

**DOI:** 10.1155/2020/4594606

**Published:** 2020-06-18

**Authors:** Renita M. Martis, Luis J. Knight, Paul J. Donaldson, Julie C. Lim

**Affiliations:** ^1^Department of Physiology, School of Medical Sciences, University of Auckland, Auckland, New Zealand; ^2^New Zealand National Eye Centre, University of Auckland, Auckland, New Zealand

## Abstract

The cystine/glutamate antiporter (system *x*_c_^−^) is composed of a heavy chain subunit 4F2hc linked by a disulphide bond to a light chain xCT, which exchanges extracellular cystine, the disulphide form of the amino acid cysteine, for intracellular glutamate. *In vitro* research in the brain, kidney, and liver have shown this antiporter to play a role in minimising oxidative stress by providing a source of intracellular cysteine for the synthesis of the antioxidant glutathione. *In vivo* studies using the xCT knockout mouse revealed that the plasma cystine/cysteine redox couple was tilted to a more oxidative state demonstrating system x_c_^−^ to also play a role in maintaining extracellular redox balance by driving a cystine/cysteine redox cycle. In addition, through import of cystine, system x_c_^−^ also serves to export glutamate into the extracellular space which may influence neurotransmission and glutamate signalling in neural tissues. While changes to system x_c_^−^ function has been linked to cancer and neurodegenerative disease, there is limited research on the roles of system x_c_^−^ in the different tissues of the eye, and links between the antiporter, aging, and ocular disease. Hence, this review seeks to consolidate research on system x_c_^−^ in the cornea, lens, retina, and ocular humours conducted across several species to shed light on the *in vitro* and *in vivo* roles of xCT in the eye and highlight the utility of the xCT knockout mouse as a tool to investigate the contribution of xCT to age-related ocular diseases.

## 1. Introduction

The cystine/glutamate exchanger, referred to as system x_c_^−^, is responsible for the Na^+^-independent electroneutral exchange of cystine and glutamate [1]. It is a member of the heteromeric amino acid transporter family and is composed of a heavy subunit 4F2hc (SLC3A2) that is involved in the trafficking of the heterodimer to the plasma membrane, and a light subunit xCT (SLC7A11), which is responsible for the exchange of extracellular cystine for intracellular glutamate [2]. Whereas 4F2hc is a subunit common to several amino acid transport systems [3], xCT is unique for cystine/glutamate exchange.

There has been considerable interest in the role of system x_c_^−^, especially in pathological conditions such as cancer [4], microbial infection [5], neurodegenerative disease [4, 6], and more recently ocular disease [7]. In the eye, oxidative damage and the subsequent depletion of antioxidants underlie several major eye diseases such as age-related nuclear (ARN) cataract, age-related macular degeneration (ARMD), and diabetic retinopathy [4]. The antioxidant glutathione is a principal antioxidant in many ocular tissues and is synthesised from the amino acids cysteine, glutamate, and glycine. In other tissues, system x_c_^−^ has been shown to be a major source of cysteine, the rate limiting amino acid required for GSH synthesis, suggesting that upregulation of system x_c_^−^ could help to restore cysteine and/or GSH levels, preventing or slowing down the progression of ocular pathologies initiated by oxidative damage [8–15]. In studies of mouse retinal ganglion cells, nitric oxide and reactive oxygen species were shown to upregulate system x_c_^−^ via an increase in xCT mRNA expression and protein levels which resulted in a concomitant increase in GSH [16]. However, upregulation of system x_c_^−^ was also associated with an increased release of glutamate. If the Müller cell function is compromised as in the case of diabetes [17], excess glutamate will not be removed, and upregulation of system x_c_^−^ would be expected to be particularly detrimental to ganglion cells and visual function due to increased glutamate excitotoxicity. Thus, the potential utility of system x_c_^−^ in influencing antioxidant homeostasis and glutamate signalling pathways in ocular tissues is particularly relevant.

The aim of this review is to consolidate the role of system x_c_^−^ in the different tissues of the eye and to determine whether targeting system x_c_^−^ can be used to restore intracellular and extracellular redox balance in the aging eye.

## 2. Cystine/Glutamate Antiporter Structure and Function

### 2.1. Structure

**Figure 1:**
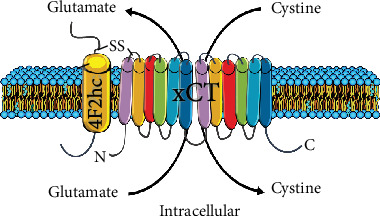
Structure of system x_c_^−^. System x_c_^−^ is made up of two subunits: the heavy chain 4F2hc and the light chain xCT linked by a disulphide bond (SS). 4F2hc spans the membrane once and is responsible for anchoring the antiporter to the membrane. xCT contains 12 transmembrane domains with the C- and N-termini located intracellularly. The main function of xCT is to import cystine in exchange for glutamate at a molar ratio of 1 : 1.

System x_c_^−^ is composed of two chains linked by a disulphide bridge; the ubiquitous heavy chain (4F2hc) subunit, which anchors the antiporter to the membrane, and the light chain (xCT) subunit (Figure 1). The 4F2hc subunit is a type II glycoprotein with a single transmembrane domain, an intracellular NH_2_ terminus, and a molecular weight of approximately 85 kDa [18]. The xCT light chain subunit has 12 putative transmembrane domains with the N- and C-termini located intracellularly; it is not glycosylated and has a predicted molecular mass of approximately 55 kDa [2]. It confers transport specificity and mediates the exchange of extracellular cystine for intracellular glutamate at a molar ratio of 1 : 1. Transport of substrates is Na^+^-independent and Cl^−^-dependent [19]. Since extracellular cystine levels are higher than intracellular levels, cystine is transported into the cell, while the higher concentrations of intracellular glutamate relative to extracellular glutamate result in export of glutamate from the cells [10]. In addition to the uptake of cystine, recent evidence shows that system x_c_^−^ is also able to uptake cystathionine, a precursor amino acid of cysteine [20].

### 2.2. Function of System x_c_^−^ in Other Tissues

**Figure 2:**
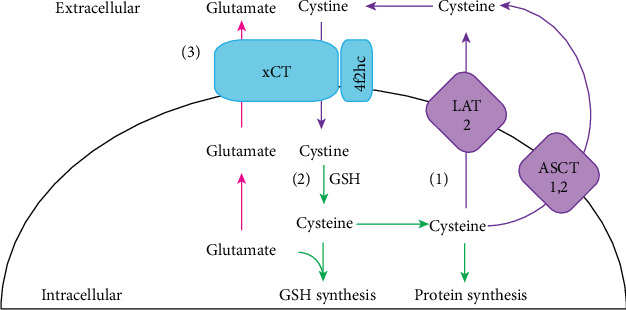
Proposed roles of system x_c_^−^. Three main roles have been proposed for system x_c_^−^. (1) Excess cysteine is exported out of the cell via alanine-serine-cysteine transporter (ASCT) 1 and 2 or the system L amino acid transporter (LAT) 2 where it is oxidised to cystine and then taken up by xCT to maintain extracellular cysteine/cystine redox balance. (2) Cystine taken up by xCT is then reduced to cysteine, which is used for either GSH or protein synthesis. (3) Glutamate is transported out of the cell by xCT, which takes on added significance in neuronal tissues as it represents a nonvesicular route of release through which glutamate can potentially participate in neuronal signalling.

While there are several *in vitro* studies to elucidate the role of xCT in cell lines, the generation of an xCT knockout (KO) mouse by Sato et al., using gene targeting strategy, enabled investigators to study the role of xCT *in vivo* [13]. Although xCT KO mice were healthy in appearance, fertile, and lived a normal lifespan [21], distinct biochemical changes were observed, which, when complemented with *in vitro* studies, have led to the identification of three roles for xCT. These are (1) uptake of cystine to maintain extracellular cysteine/cystine redox balance, (2) uptake of cyst(e)ine for GSH synthesis, and (3) export of glutamate (Figure 2). In the next section, explanations of each of these roles based on findings from *in vitro* and *in vivo* studies are detailed.

#### 2.2.1. Maintaining Extracellular Cysteine/Cystine Redox Balance

Cysteine and its disulphide cystine is the predominant extracellular thiol/disulphide couple and is used as an indicator of the extracellular redox state [22]. In humans, an oxidative shift of the plasma cysteine/cystine ratio occurs in which plasma cystine concentrations increase relative to cysteine levels with increasing age. This leads to an oxidative shift of approximately 0.2 mV per year [23]. An oxidative shift of the cysteine/cystine ratio is also seen in the plasma of patients with cancer [24], cardiovascular disease ([25]), atherosclerosis [26], diabetes [27], and lung pathologies [28].

System x_c_^−^ contributes to the maintenance of the extracellular cysteine/cystine cycle via uptake of cystine via xCT, intracellular reduction of cystine to cysteine, and the export of cysteine that is surplus to intracellular requirements out of the cell where it is then rapidly oxidised back to cystine. The importance of system x_c_^−^ in maintaining extracellular cysteine/cystine redox balance *in vivo* was first demonstrated by [13]. It was revealed that eight-week-old xCT KO mice exhibited similar plasma cysteine levels to age-matched wild-type (WT) mice, but plasma cystine concentrations in xCT KO mice were significantly increased relative to WT. Sato et al. [13] also calculated the plasma cysteine/cystine redox potential of WT (-100 mV) and xCT KO (-89 mV) mice and found an oxidative change of 11 mV, indicative of an oxidative shift of the cysteine/cystine couple. This is reminiscent of the oxidative shift of the plasma cysteine/cystine ratio in aging individuals [23], implying that aging may be accelerated in the xCT KO mouse.

#### 2.2.2. GSH Synthesis

Another important role of system x_c_^−^ is the uptake of cyst(e)ine for GSH synthesis. Sato et al. [13] observed that cultured fibroblasts derived from xCT KO mice failed to survive as they were unable to maintain the intracellular cysteine levels required for the synthesis of GSH [13]. The meninges and other cell types such as the astrocytes, glial cells, lens epithelial cells, and retinal pigment epithelial cells have also been implicated to utilise xCT to import cystine for GSH synthesis [9–11, 14]. Furthermore, under conditions of oxidative stress or disease states, increased expression and upregulation of xCT activity is observed by cells to maintain GSH levels [8, 12, 15].

Interestingly, while *in vitro* studies revealed that xCT is a major source of cyst(e)ine for GSH synthesis, *in vivo* studies indicate that xCT may not be solely responsible for maintaining GSH levels and that other compensatory mechanisms exist. In the xCT KO mouse, GSH content in the cerebrum, cerebellum, hippocampus, striatum, liver, kidney, thymus, spleen, lung, heart, and pancreas was similar to that of the WT, indicating that in the absence of xCT, these tissues were able to maintain GSH levels via alternative mechanisms [6, 13, 20, 29]. Sato et al. [13] found that cultured fibroblasts derived from xCT KO mice did not survive. However, the addition of the reducing agents *β*-mercaptoethanol (*β*ME) or N-acetylcysteine resulted in an increase in intracellular cysteine and GSH levels, allowing for cell growth and survival. The survival of these cells despite the absence of xCT was attributed to the availability of extracellular cysteine and its intracellular accumulation through neutral amino acid transporters. These alternative pathways may include synthesis of cysteine via the transsulfuration pathway, uptake of cystine via b^0,+^AT [30], or via the excitatory amino acid transporters (EAAT) 1-3 [31], the alanine-serine-cysteine transporters (ASCT) 1 and 2, or system L amino acid transporters (LAT) 2 [32].

#### 2.2.3. Control of Extracellular Glutamate Levels

More recent evidence suggests that system x_c_^−^ plays a role in the nonvesicular, calcium- (Ca^2+^-) independent release of glutamate, which is of particular significance to neuronal tissues such as the brain and the retina. In the brain, the xCT release of extracellular glutamate is observed in the hippocampus and striatum, where studies have demonstrated that glutamate levels in these regions decreased by 60-70% in xCT KO mice when compared to the WT [6, 29]. In the retina, Ca^2+^-independent glutamate release was observed when toad retinas were incubated in a Ca^2+^-depleted medium, and a glutamate-mediated response of the inner retina continued during conditions expected to block the entry of Ca^2+^ into their presynaptic terminals [33]. In the rat retina, Hu et al. [34] demonstrated a Ca^2+^-independent release of glutamate via xCT suggesting a role for xCT in neurotransmission and glutamate signalling [34]. Further details of the roles of xCT in the retina can be found in Section 4.3.

## 3. Expression of xCT in Ocular Tissues

**Figure 3:**
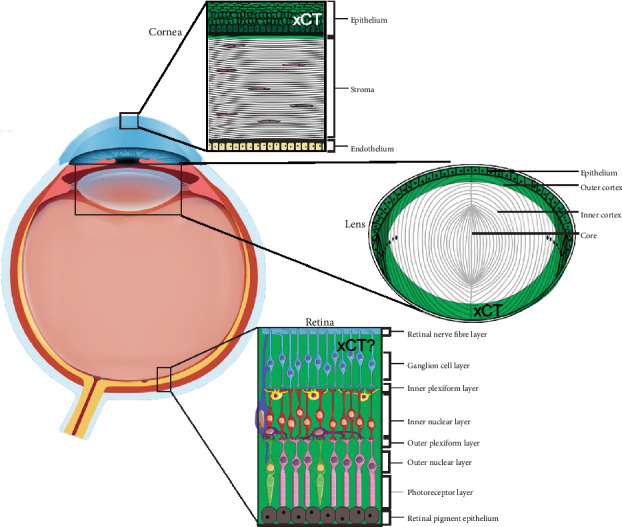
Schematic highlighting xCT mapping studies in ocular tissues. xCT labelling detected in the cornea [35, 37, 38], lens [10, 35, 40, 41], and retina [34, 35] collated from various studies. xCT labelling (*green*) was detected in the corneal epithelium but absent from the stroma and endothelium. In the lens, xCT (*green*) labelling in the mouse was detected in the epithelium and cortical fibres. However, in other species such as rat and human, xCT labelling could also be detected in the deeper lying fibre cells (not shown). In the retina, xCT labelling was detected in all the cell layers. However, these labelling patterns at least in the mouse retina could not be validated due to the lack of specificity of commercial xCT antibodies in neuronal tissue (highlighted by the “?”). The diagram was adapted from [35]. Retinal elements were adapted from Servier Medical Art, which is licensed under a Creative Commons Attribution 3.0 Unported License.

The specific localisation of xCT in tissues, in particular neural tissues such as the brain and retina, has been difficult to evaluate, due to the reliability of specific antibodies recognizing xCT by immunohistochemical methods [35, 36]. As such, complementary techniques such as PCR and western blotting have been used in combination with an xCT KO mouse or xCT antigenic peptide as a control to confirm the specificity of xCT expression. xCT has been identified in ocular tissues such as the cornea [37, 38], ciliary body [39], lens [10, 40, 41], and retina [16, 34, 42, 43] of various species. A summary of these expression studies in the various tissues of the eye of different species is listed in Tables 1 and 2. In addition, a schematic of xCT localisation in the different mouse ocular tissues where labelling patterns have been verified using an xCT KO mouse is summarised in Figure 3.

### 3.1. Cornea

While RNA and western blot studies have confirmed expression of xCT in the mouse cornea [35], immunohistochemistry was used to visualize the localisation of xCT in the different cellular layers of the cornea. Langford et al. showed that xCT expression in the human cornea is restricted to the corneal epithelium [37]. A similar finding was also found in the rat cornea [38] and mouse cornea [35]. In the rat and human cornea, the localisation of xCT to the epithelium coincided with the localisation of glutamate (EAATs) and glycine (GLYTs) transporters as well as the glutathione synthesising enzyme, glutamate cysteine ligase (GCL), suggesting that xCT is likely to be involved in GSH synthesis in this region of the cornea [37, 38, 44, 45]. On the other hand, the absence of xCT in the endothelium coincided with the expression of putative GSH uptake transporters; organic anion transporter 3 (OAT3) and the Na^+^-dependent dicarboxylate carrier 3. This suggests that in the endothelium, GSH accumulation is likely to occur predominantly by direct uptake of GSH from the aqueous humour [38].

### 3.2. Lens

RT-PCR and western blot studies confirmed the expression of xCT in the rat, mouse, canine, and human lens, as well as canine and human lens epithelial cells [10, 35, 40, 41, 46]. Unlike the cornea where xCT localisation was similar across species, in the lens, differences were found in the localisation of xCT in the lens of the rat and human compared to that of the mouse. In the young rat (p21 days), embryonic mouse (E16), and young human lens (16 years), xCT was localised to the epithelium and lens fibre cells, extending from the cortex to the centre of the lens [35, 40, 41]. The labelling of xCT in the epithelium and cortical fibre cells is consistent with a role for xCT in supplying cyst(e)ine for GSH synthesis. However, the localisation of xCT to the lens centre, an area not capable of protein synthesis, suggests that xCT in this region may mediate uptake of cystine where it may be locally reduced to cysteine and then used as a low molecular mass antioxidant to protect fibre cells from oxidative damage [40, 41, 47]. Unlike the embryonic mouse lens, in the young mouse lens (p21), xCT was seen only in the epithelium and cortical fibre cells, and this labelling pattern was verified in the xCT KO mouse [35]. This change in labeling pattern was also observed in an older human lens (64 yrs) where xCT was absent from the epithelium and core. Together in the mouse and human lens, the absence of xCT in the core suggests that with advancing age, xCT may be posttranslationally modified and therefore undetectable in the lens centre. It is unclear at this stage how these posttranslational changes to the xCT protein in the human and mouse lens affect xCT function in the core.

### 3.3. Retina

xCT mRNA has been identified in the human retina, and xCT protein has been detected in the retina and various retinal cells in the rat, canine, and mouse [10, 15, 16, 34, 35, 42, 48, 49]. However, the lack of specificity of xCT labelling in brain tissue [36] has raised concerns about the specificity of xCT labelling in retinal tissue. Attempts to localise xCT in both the mouse and rat retina have revealed similar labelling patterns, with xCT being present in both the inner and outer synaptic plexiform layers, the photoreceptor cell bodies of the outer nuclear layer, and the retinal ganglion cell layer [16, 34, 42]. Hu et al., [34] used electron microscopy to show that xCT was colocalised with the ribbon-associated protein bassoon in the photoreceptor synapse, suggesting that xCT may have a role in mediating glutamate release from the photoreceptor presynapse. However, recent work by our group showed that xCT labelling was apparent in all the layers of the retina, in both the WT and xCT KO mouse retina, indicating that the labelling was nonspecific. While the xCT cellular localization in the mouse retina could not be confirmed [35], the xCT KO mouse can still be used as a control to infer localisation by using antibodies directed against selective substrate aminoadipic of xCT as previously conducted in rat retina and brain slices [49].

## 4. Functional Role of the Cystine/Glutamate Antiporter in Ocular Tissues

Since xCT appears to be expressed in the different tissues of the eye, a number of different methods have been used to evaluate the function of xCT in ocular tissues. This has been achieved through pharmacological inhibition of xCT by (S)-4-carboxyphenylglycine or L-glutamate, modulating xCT activity using elevated exogenous cystine [10, 34, 44, 46], or the use of an xCT KO mouse [35].  Table 3 provides a summary of *in vivo* and *in vitro* studies conducted to test the function of xCT in ocular tissues, alongside other tissues of the body. In the next section, functional studies performed to investigate the role of xCT in the cornea, lens, and retina are described.

### 4.1. Cornea

*In vitro* studies using human corneal epithelial cells demonstrated that inhibition of xCT by L-glutamate resulted in a 24% decrease in GSH levels [44]. *In vivo* studies revealed that there were no differences in cystine or cysteine levels between WT and xCT KO mice at 6 weeks of age [35]. Interestingly, GSH levels were decreased by 43% in xCT KO mice at six weeks of age but were similar between WT and xCT KO corneas at three months, six months, nine months, and 12 months of age (unpublished data). This suggests that xCT contributes to maintaining GSH levels in the young cornea but not in older mice. We have shown *in vivo* using the xCT KO mouse that xCT may also play a role in wound healing [52]. In a series of experiments conducted by our laboratory, repeated eye examinations which included rebound tonometry, slit lamp microscopy, optical coherence tomography, and fundus imaging were performed on a single cohort of WT and xCT KO mice as they aged from six weeks to 12 months. The corneas of six-week-old WT and xCT KO mice were clear, transparent, and devoid of blood vessels. As early as 3-6 months of age, both WT and xCT KO corneas developed signs of keratopathy, although the size and severity of these lesions were far greater in the xCT KO corneas than that seen in the WT corneas. The xCT KO corneas also developed signs of neovascularisation, an abnormal response, by 9 months of age [52]. During wound healing in the cornea, tumour necrosis factor alpha (TNF-*α*) is released which normally would act to upregulate xCT [53, 54]. Immune cells such as macrophages and T-cells rely on xCT through the release of TNF-*α* via the antigen-immune response for cystine uptake and subsequent GSH synthesis [54]. Since xCT KO corneas were more susceptible to external trauma than WT mice, loss of xCT may affect the TNF-*α* pathway and reduce the ability of the cornea to repair and heal itself [52].

### 4.2. Lens

A study using human lens epithelial cells showed that inhibition of xCT by L-glutamate resulted in depletion of cysteine levels by 52% and depletion of GSH by 28% [46]. However, in whole lenses, the levels of cystine and cysteine were similar between WT and xCT KO mice [35]. In canine lens epithelial cells, inhibition of xCT by (S)-4-carboxyphenylglycine resulted in a 30% decrease of cysteine levels, and with inhibition by L-glutamate a 55% decrease of cysteine levels [10], demonstrating that at least *in vitro* xCT is involved in the maintenance of cysteine and GSH levels. It should be noted that there was never a complete depletion of cysteine or GSH suggesting other mechanisms are likely to be involved in maintaining GSH homeostasis. *In vivo* studies using the xCT KO mouse will be instrumental in clarifying the role of xCT in the lens particularly since the importance of GSH in the lens is well documented [55–58].

### 4.3. Retina

*In vitro* studies have demonstrated the importance of xCT in cystine uptake and maintenance of GSH levels in mouse and canine retinal pigmented epithelial (RPE) cells, the rat Müller cells, and mouse retinal ganglion (RGC) cells ([50, 10, 15, 16, 51, 59, 60]). Several studies have demonstrated that induction of xCT expression by agents such as nitric oxide, or diethyl maleate, resulted in increased cystine uptake and increased GSH levels in whole cultured retinas from the mouse and rat, isolated rat Müller cells, rat RGC cells, and human RPE cells [15, 16, 51, 61]. Conversely, inhibition of the transcription factor Nrf2, which reduces xCT expression, led to a reduction in GSH levels in the cultured Müller cells [60]. Furthermore, xCT expression decreases in rat RPE cells incubated in high glucose and in retina tissue of streptozotocin-induced diabetic rats. In the case of the diabetic rats, three weeks after the induction of diabetes, immunohistochemistry revealed xCT expression to be significantly decreased in the inner nuclear layer and ganglion cell layer with GSH levels decreased by ~24% indicating that xCT function is affected in diabetic rats [51].

While it appears that xCT plays a role in maintaining GSH levels in the retina, xCT also plays a role in the export of glutamate that is distinct from the traditional Ca^2+^-dependent vesicular release of glutamate for neurotransmission. Hu et al. [34] first demonstrated this using the cation channel probe, agmatine. Under light conditions, glutamate released via xCT in the retinas incubated in cystine induced the release of glutamate, which activated postsynaptic ON bipolar mGluR6 receptors resulting in the closure of nonselective cation channels. The ultrastructural localisation of xCT to the ribbon synapse, and the functional modulation of postreceptoral neurons, may indicate a role for xCT in glutamate neurotransmission that complements the established vesicular-mediated glutamate release [34]. In the hippocampus, the Ca^2+^-independent release of glutamate reduces the number of postsynaptic inotropic (AMPA) receptors [62], but whether a similar function takes place in the retina is unclear. Alternatively, xCT may be involved in maintaining basal levels of glutamate in the outer retina which modulates the sensitivity and clustering of the postsynaptic glutamate receptors [63, 64].

An interesting aspect of cystine/glutamate exchange by xCT is that while upregulation of xCT may reduce oxidative stress through enhanced uptake of cystine and synthesis of GSH, the concomitant export of glutamate will lead to increased levels of glutamate in the extracellular milieu and excitotoxicity, if not rapidly cleared from the extracellular milieu by high-affinity excitatory amino acid transporters (EAATs) [65]. In the primary mouse Müller cells, the function of system x_c_^−^ increases dramatically under oxidative stress; however, the activity of the GLAST transporter (EAAT1) remains remarkably robust even with considerable oxidative insults [48]. The *in vivo* interplay between xCT and the EAATs in regard to neurotransmission in the retina needs to be further explored and should be an area of interest for future studies.

### 4.4. Ocular Humours

Since Sato et al. [13] previously showed that xCT KO mice exhibit an oxidative shift in the cysteine/cystine ratio in the plasma, our group measured cysteine and cystine levels in the aqueous and vitreous humour to determine whether a similar oxidative shift was apparent in the ocular humours [35]. While cysteine levels were similar in the aqueous and vitreous humour between 6 week xCT KO and WT mice, cystine levels were significantly higher in the aqueous humour, but not the vitreous humour of the xCT KO mouse compared to the WT [35]. These results suggest that the loss of xCT results in an oxidative shift of the aqueous humour which has the potential to expose the tissues of the eye which interface with the aqueous humour, such as the corneal endothelium, the trabecular meshwork, the iris, the ciliary body, and the anterior lens, to a heightened oxidative environment. Whether this leads to oxidative damage in these tissues and subsequent pathologies will be of interest in the future.

## 5. Cystine/Glutamate Antiporter and Vision

The contribution of xCT to visual function was examined by performing optomotor tests on adult (16-20 weeks) and aged (19-23 months) WT and xCT KO to evaluate their visual acuity. While an age-related decline in visual acuity was reported between genotypes, loss of xCT did not affect visual acuity between age-matched WT and xCT KO mice [21]. A similar finding was also found for sensorimotor function, with suggestion that the age-related decline in visual acuity might have contributed, at least partly, to the age-related effects observed in other behavioural tests evaluating motor function [21]. Further studies evaluating extracellular glutamate levels in the retina and neurotransmission in xCT KO mice will help to better define the role of xCT in visual function.

## 6. Conclusions and Future Studies

With an increasing aging population, research efforts have focused on identifying the molecular mechanisms involved in maintaining normal ocular structure, function, and physiology in order to gain a better understanding of how this may change with advancing age and/or disease. The xCT KO mouse may represent a unique model of accelerated aging through altering the extracellular redox environment. Genetic deletion of xCT results in an oxidative shift of the plasma reminiscent of that seen in aging humans, which manifests in the eye as an oxidative shift of the aqueous humour. The xCT KO mouse serves as a valuable tool for not only dissecting the role of xCT in the different tissues of the eye but also determining how a heightened oxidative environment affects the function and health of the lens and retina. Finally, the xCT KO mouse may prove to be a useful model towards exploring therapeutic strategies for restoring cysteine/cystine redox balance and delaying or preventing the onset of aging in the eye.

## Figures and Tables

**Table 1:** Summary of xCT expression in ocular tissues.

	Species	Tissue	Reference
PCR (RNA/DNA)	Mouse	Cornea^∗^	[35]
		Lens^∗^	
		Retina^∗^	
		RGC	[16]
	Rat	Lens	[40]
		RPE cells	[50]
		Retina	[34]
		Müller cells	[15]
	Human	Lens	[41]
		Retina	[10]
		RPE cells	
	Canine	LEC	[10]
		RPE cells	

Western blotting	Mouse	Cornea∗	[35]
		Lens^∗^	
		Retina^∗^	
		RGC	[16]
		Müller cells	[48]
	Rat	Lens	[40]
		Retina	([51, 34])
		Müller cells	[15]
	Human	Lens	[41]
		LEC	[46]
		RPE	[10]
	Canine	LEC	[10]
		RPE	

^∗^Labelling specificity verified using an xCT KO mouse. RPE: retinal pigment epithelium; LEC: lens epithelial cells; RGC: retinal ganglion.

**Table 2:** Summary of xCT localisation in ocular tissues.

	Species	Tissue	Localisation	Reference
Immunohistochemistry	Mouse	Cornea	Epi^∗^	[35]
		Lens	Epi, OC^∗^	
		Embryonic lens	Epi, OC, IC, Core	
		Retina	No specific labelling^∗^	
			All layers	[42, 16]
	Rat	Cornea	Epi	[38, 45]
		Lens	Epi, OC, IC, Core	[40]
		Retina	IPL, OPL, Müller cells	[34, 49, 15]
	Human	Cornea	Epi	[37]
		Lens	Young donor: Epi, OC, IC, core	[41]
			Old donor: OC, IC	
		Retina	RGC	[16]
	Canine	Lens	Epi, OC	[10]

^∗^Labelling specificity verified using an xCT KO mouse. Epi: epithelium; OC: outer cortex; IC: inner cortex; IPL: inner plexiform layer; OPL: outer plexiform layer; RGC: retinal ganglion cells.

**Table 3:** Summary of xCT function.

Function tested	*In vivo* or *in vitro*	Where	Results	Reference
Maintaining cysteine/cystine redox balance	*In vivo*	Plasma, aqueous humour, vitreous humour	Loss of xCT resulted in increased cystine levels by 2 folds while cysteine levels remained the same	[35, 13]

GSH synthesis	*In vitro*	HCEC, HLEC, rat retinal cell culture, ARPE-19, rat RPE, retinal epithelial cells, rat Müller cells, mouse fibroblasts	GSH levels decreased as a result of inhibition of xCT	[51, 16, 10, 46, 44, 15]
		Mouse RPE	Increased xCT expression results in increased GSH levels	[59]
	*In vivo*	Cerebrum, cerebellum, hippocampus, striatum, liver, kidney, thymus, spleen, lung, heart, and pancreas	No change in GSH levels between WT and xCT KO mice	[29, 20, 6, 13]

Control of extracellular glutamate levels	*In vitro*	Hippocampus	Glutamate released via xCT regulates glutamatergic synapse strength by reducing the number of postsynaptic AMPA receptors	[62]
		Retina	Glutamate release via xCT is distinct from the traditional Ca^2 +^-dependent vesicular release of glutamate	[34]
	*In vivo*	Striatum, hippocampus	Extracellular glutamate levels decrease in the xCT KO mouse when compared to WT	[29, 6]

HCEC: human corneal epithelial cells; HLEC: human lens epithelial cell; ARPE-19: human retinal epithelial cell; RPE: retinal epithelial cell.
